# Predictive Ability of a Clinical-Genetic Risk Score for Venous Thromboembolism in Northern and Southern European Populations

**DOI:** 10.1055/s-0041-1729626

**Published:** 2021-07-08

**Authors:** Eduardo Salas, Maria Farm, Sara Pich, Liselotte Onelöv, Kevin Guillen, Israel Ortega, Jovan P. Antovic, Jose Manuel Soria

**Affiliations:** 1 Scientific Department, Gendiag, c/ Lepant, 141-4-1, 08013 Barcelona, Spain; 2 Institute for Molecular Medicine and Surgery and Department of Clinical Chemistry, Karolinska Institutet and Karolinska University Hospital, Stockholm, Sweden; 3 Genomic of Complex Diseases, Institut d'Investigació Sant Pau (IIB-Sant Pau), Barcelona, Spain

**Keywords:** venous thromboembolism, genetic, risk score, primary prevention, anticoagulation, pulmonary embolism, deep vein thrombosis

## Abstract

Venous thromboembolism (VTE) is a complex, multifactorial problem, the development of which depends on a combination of genetic and acqfiguired risk factors. In a Spanish population, the Thrombo inCode score (or TiC score), which combines clinical and genetic risk components, was recently proven better at determining the risk of VTE than the commonly used model involving the analysis of two genetic variants associated with thrombophilia: the Factor V Leiden (F5 rs6025) and the G20210A prothrombin (F2 rs1799963).

The aim of the present case–control study was to validate the VTE risk predictive capacity of the TiC score in a Northern European population (from Sweden).

The study included 173 subjects with VTE and 196 controls. All were analyzed for the genetic risk variants included in the TiC gene panel. Standard measures —receiver operating characteristic (ROC) area under the curve (AUC), sensitivity, specificity, and odds ratio (OR)—were calculated.

The TiC score returned an AUC value of 0.673, a sensitivity of 72.25%, a specificity of 60.62%, and an OR of 4.11. These AUC, sensitivity, and OR values are all greater than those associated with the currently used combination of genetic variants. A TiC version adjusted for the allelic frequencies of the Swedish population significantly improved its AUC value (0.783).

In summary, the TiC score returned more reliable risk estimates for the studied Northern European population than did the analysis of the Factor V Leiden and the G20210A genetic variations in combination. Thus, the TiC score can be reliably used with European populations, despite differences in allelic frequencies.

## Introduction

Venous thromboembolism (VTE)—primarily deep vein thrombosis (DVT) and pulmonary embolism (PE)—is a common disorder that affects some 0.2% of the population annually. Mortality rates reach between 5% (DVT) and 33% (PE) within the first months of diagnosis. 1 2 VTE is thought to be the leading cause of preventable hospital mortality. 3 Unfortunately, survivors of VTE are at risk of long-term complications, such as recurrence, postthrombotic syndrome, and pulmonary hypertension. 1 4 5 VTE recurs in 20 to 30% of patients within 5 years. 6 7 It is, therefore, a considerable public health problem with a large economic burden. 8 9 10

VTE is a complex, multifactorial problem, the development of which depends on a combination of genetic and acquired risk factors (with the former responsible for some 60% of the total risk). 11 Until recently, the risk of VTE was determined by testing for the Factor V Leiden (F5 rs6025) and the G20210A prothrombin (PT) (F2 rs1799963) genetic variants only (hereinafter the F5L + F2 combination). This has been challenged, however, by the Thrombo inCode (TiC) score, a clinical/genetic algorithm for assessing the risk of VTE developed by Soria et al. 12 As well as taking into account several clinical variables, the TiC score includes low-frequency genetic variants with high odds ratios (ORs) for thrombosis, as well as common risk alleles with low ORs. Compared with the F5L + F2 combination, this risk score returned a significantly higher area under the receiver operating characteristic (ROC) curve (AUC) for a population from Sant Pau in Spain (0.677 vs. 0.575; *p* < 0.001). It also showed good reclassification capacity and had a high integrated discrimination index. 12

The TiC score takes into account the single-nucleotide polymorphisms (SNPs) F2 rs1799963, F5 rs6025 (Factor V Leiden), F5 rs118203905 (Factor V Cambridge), F5 rs118203906 (Factor V Hong-Kong), F12 rs1801020 (in the gene for Factor XII), SERPINC1 rs121909548 (Antithrombin Cambridge II), and SERPINA10 rs2232698 (in the gene that codes for protein Z-dependent protease inhibitor), along with SNPs in the ABO gene that predispose one to blood type A1 (ABO rs8176719, ABO rs7853989, ABO rs8176743, and ABO rs8176750), and a protective variant F13 rs5985 (in the gene that codes for Factor XIII). The addition of genetic variants from genome-wide association studies, such as F11 rs2036914 (in the gene for Factor XI) and fibrinogen gamma gene (FGG) rs2066865 (in the gene for the fibrinogen γ chain), did not improve the results obtained. 12

One of the potential concerns with genetic risk scores (GRS), however, is that the magnitude and direction of allelic effects can differ between populations. An example is the north European aggregation of F5 rs6025. 13 The main aim of the present work was to validate the VTE risk predictive capacity of the TiC score in subjects from a Northern European country (Sweden), among which the frequency of at least F5 rs6025 is higher than in southern Europe.

## Methods

### Study Population

This case–control study was performed at the Department of Molecular Medicine and Surgery at the Karolinska Institute (Stockholm, Sweden), in a Swedish population whose members had experienced a VTE, and who had consequently been tested for thrombophilia. All consecutive adult patients with samples examined by the Karolinska University Hospital Coagulation Laboratory between 2014 and 2016 for inherited thrombophilia were invited to participate. After written informed consent was obtained, only those who fulfilled the clinical criteria for thrombophilia testing were included, that is, having suffered a first provoked or unprovoked VTE (DVT or PE) before the age of 50. As family history is usually used to identify the subjects at high risk of VTE, we have forced the controls to have a similar family history of VTE than the cases. The final study population was 173 unrelated patients; 196 apparently healthy persons were recruited as controls. To avoid genetic stratification, the members of both the case and control groups were all recruited from central Sweden. None of the patients or controls had been prescribed VTE prophylactic treatment.

A medical history was obtained for each subject, including their acquired risk factors for VTE. A diagnosis of DVT in the lower limbs was established objectively by ultrasonography or ascending venography. PE was diagnosed by computed tomography, pulmonary angiography, or ventilation–perfusion lung scintigraphy. A subject's family history was considered positive if at least one first-degree family member had suffered a VTE.

### DNA Extraction and Genetic Analysis

DNA was extracted from leukocytes in ethylenediaminetetraacetic acid-treated whole blood by digestion and selective precipitation with ethanol in an automated QiaCube system using the QIAamp DNA-blood mini Kit (Qiagen, Düsseldorf, Germany) following the manufacturer's instructions. Extracts were stored at –20°C until use.

Table 1 shows a comprehensive summary of the genes examined. The prothrombotic genetic variables associated with the TiC score were genotyped using the Thrombo inCode kit (GEN inCode, Barcelona, Spain).

**Table 1:** Genetic variants analyzed across the three genetic risk scores examined

SNP	Gene	TiC*GRS ONLY	F5L + PT	TiC*GRS ONLY +F11 + FGG
rs6025	F5 Leiden	X	X	X
rs118203905	F5 Hong Kong	X		X
rs118203906	F2 Cambridge	X		X
rs1799963	F2 G20210A	X	X	X
rs8176719	ABO A1	X		X
rs7853989	ABO A1	X		X
rs8176743	ABO A1	X		X
rs8176750	ABO A1	X		X
rs1801020	F12	X		X
rs5985	F13	X		X
rs2232698	Serpin A10 a	X		X
rs121909548	Serpin C1 b	X		X
rs2036914	F11			X
rs2066865	Fibrinogen			X

Abbreviations: FGG, fibrinogen gamma gene; GRS, genetic risk score; PT, prothrombin; SNP, single-nucleotide polymorphism; TiC, Thrombo inCode.

a Protein Z-dependent protease inhibitor.

b Antithrombin.

The F11 rs2036914 and FGG rs2066865 variants were genotyped using the TaqMan genotyping assay from Life Technologies (Foster City, California, United States) on the Fluidigm genotyping platform (South San Francisco, California, United States). All genetic analyses were performed at Gendiag.exe (Barcelona, Spain).

### Determining the Individual Risk of Venous Thrombosis

The individual risk for first VTE was determined using the TiC score risk algorithm. This uses the results of the genetic analysis associated with the score alongside clinical variables recognized as risk factors of VTE: age, gender, body mass index (BMI), smoking habit, presence of type II diabetes, and a family history of thrombosis ( Table 2 ). For women, it also includes pregnancy and treatment with prothrombotic hormonal contraceptives. 2 7 14 An overall risk value is then determined. 12

**Table 2:** Clinical and genetic variants included in the different algorithms studied

Variable	TiC*Clinical ONLY	F5L + F2	F5L + F2 + TiC*Clinical ONLY	TiC*GRS ONLY	TiC	TiC*GRS ONLY +F11 + FGG	TiC + F11 + FGG	TiC*GRS ONLY-MOD	TiC-MOD
F5 rs6025		X	X	X	X	X	X	X	X
F5 rs118203905				X	X	X	X	X	X
F5 rs118203906				X	X	X	X	X	X
F2 rs1799963		X	X	X	X	X	X	X	X
ABO rs8176719				X	X	X	X	X	X
ABO rs7853989				X	X	X	X	X	X
ABO rs8176743				X	X	X	X	X	X
ABO rs1801020				X	X	X	X	X	X
F13 rs5985				X	X	X	X	X	X
SerpinA10 rs2232698				X	X	X	X	X	X
SerpinC1 rs121909548				X	X	X	X	X	X
F11 rs2036914						X	X		
FGG rs2066865						X	X		
Age	X		X		X		X		X
Gender	X		X		X		X		X
BMI	X		X		X		X		X
Smoking	X		X		X		X		X
Diabetes	X		X		X		X		X
VTE family history	X		X		X		X		X
Pregnancy	X		X		X		X		X
Use of pro-thrombotic hormonal contraceptives	X		X		X		X		X

Abbreviations: BMI, body mass index; FGG, fibrinogen gamma gene; GRS, genetic risk score; PT, prothrombin; SNP, single-nucleotide polymorphism; TiC, Thrombo inCode; VTE, venous thromboembolism.

The capacity of other GRS and clinical/GRS algorithms ( Table 2 ) to determine the risk of VTE was also examined. In Table 2 , TiC*Clinical only means that this score if form only by the clinical variables included in TiC and with the same weight. Therefore, none of the genetic variants is included. When these involved either the clinical or genetic risk components used in the TiC score, the variables therein were given the same weight as that score. In a further analysis, logistic regression was used to modify the weight assigned to the different genetic variants in the original Spanish population, to reflect the allelic frequencies of the cases and controls in the Swedish population.

Finally, the results obtained for the Swedish population were compared with those of the Spanish population. 12

The study protocol was approved by the Regional Research Ethics Committee, Stockholm, Sweden (EPN2014/987–31/1).

### Statistical Analysis

The predictive capacity of the risk scores was evaluated using the area under the ROC curve (AUC; larger values indicate better discrimination). 15 The DeLong test was used to compare the AUC values of the different GRS and risk algorithms. Optimal cutoffs for each were calculated from the ROC data using the Youden Index.

Standard measures—sensitivity, specificity, OR and positive and negative likelihood ratios (LR + , LR–) 16 —were calculated and compared using MedCalc v.18.6 software ( http://www.medcalc.org ; 2018), which implements several methods for each measure. Briefly, sensitivity and specificity were compared using the McNemar test, likelihood ratios were compared using the chi-squared test. Age and BMI were compared using the Mann–Whitney U test, and proportions using the chi-squared test.

## Results

Table 3 shows the distribution of the clinical risk factors. As expected, the known risk factors of smoking, BMI, gender, and age differed significantly between cases and controls. The fact that there were more women in the control group probably contributed to the nonsignificant contribution of other risk factors such as pregnancy and procoagulant hormonal contraceptives. Among the 173 patients with VTE, 56 of them (32.37%) were provoked.

**Table 3:** Clinical characteristics of the study population

		All	Cases	Controls	
		*n* = 369	*n* = 173	*n* = 196	*p* -Value
Age a	Median	39.0 [37.0;41.0]	41.0 [38.1;43.9]	33.0 [30.0;36.0]	0.018
Gender b	Female	252 (68.3)	95 (54.9)	157 (80.1)	<0.0001
	Male	117 (31.7)	78 (45.1)	39 (19.9)	
BMI b	Median	24.8 [24.2;25.5)	26.5 [25.5;27.1]	24.1 [23.4;24.5]	<0.0001
Smoking b	Yes	36 (9.8)	24 (13.9)	12 (6.1)	0.0119
PHC b		*n* = 176	*n* = 71	*n* = 105	
	Yes	30 (8.1)	17 (9.8)	13 (6.6)	0.2615
Diabetes b	Yes	15 (4.1)	9 (5.2)	6 (3.1)	0.3098
Family history of VTE b	Yes	170 (46.6)	74 (43.8)	96 (49.0)	0.3183
Pregnancy b	Yes	76 (20.59)	24 (13.87)	52 (26.5)	0.0028

Abbreviations: BMI, body mass index; CI, confidence interval; PHC, procoagulant hormonal contraceptive; VTE, venous thromboembolism.

a Expressed as mean [95% CI].

b Expressed as *n* (%).

Table 4 shows the prevalence of the VTE risk alleles among the case subjects and controls. The risk alleles in the A1 ABO blood group, SERPINA10 rs2232698 and FGG rs2066865, were significantly more frequent in case subjects than in controls.

**Table 4:** Presence of risk alleles in the studied Swedish population

Gen	Cases a b	Controls a b	*p* -Value
	*n* = 173	*n* = 196	
ABO c	102 (58.96)	79 (40.31)	0.0004
F12	75 (43.35)	92 (46.94)	0.48899
Serpin A10	4 (2.31)	0	0.0326
Serpin C1	0	0	
F5 d	40 (23.12)	35 (17.86)	0.2108
F13	69 (39.88)	96 (48.98)	0.0832
F2	12 (6.93)	7 (3.57)	0.1454
F11	142 (82.08)	163 (83.16)	0.7848
FGG	99 (57.22)	87 (44.39)	0.0140

Abbreviation: SNP, single-nucleotide polymorphism.

a Expressed as *n* (%).

b With at least one risk allele.

c With at least one allele for A1 ABO subgroup.

d With at least one risk allele for any of the reference SNP cluster IDs (rs) analyzed.

Table 5 shows the prognostic characteristics of all the GRS and risk algorithms. The TiC score (TiC) returned a higher AUC value than the F5L + F2 combination (0.673 vs. 0.537; *p* < 0.0001). Standard accuracy measures were calculated at the cutoff yielded by the Youden index. The TiC score had a higher sensitivity than F5L + F2 (74.57 vs. 28.90: *p* < 0.0001) and a better LR+ (1.33 vs. 1.83; *p* < 0.0001) and LR– (0.46 vs. 0.91; *p* < 0.0001); however, it returned a lower specificity score (60.62 vs. 78.24; *p* < 0.001). These results for the Swedish population are very similar to those obtained for the Spanish 12 population.

**Table 5:** Prognostic characteristics of the different algorithms

Panel A Prognostic characteristics									
	TiC*Clinical ONLY	F5L + F2	F5L + F2+ TiC*Clinical ONLY	TiC*GRS*ONLY	TiC	TiC*GRS ONLY + F11 + FGG	TiC + F11 + FGG	TiC*GRS ONLY MOD	TiC MOD
AUC a	0.576 [0.523–0.627]	0.537 [0.484–0.589]	0.585 [0.532–0.636]	0.588 [0.536–0.639]	0.673 [0.622–0.721]	0.608 [0.556–0.659]	0.679 [0.629–0.727]	0.636 [0.584–0.685]	0.783 [0.737–0.824]
*p* -Value b	0.0117	0.1069	0.0044	0.0021	<0.0001	0.0002	<0.0001	<0.0001	<0.0001
Sensitivity	75.14 [68.0–81.4]	28.90 [22.3–36.3]	77.46 [70.5–83.5]	74.57 [67.4–80.9]	72.25 [64.9–78.8]	64.74 [57.1–71.8]	74.57 [67.4–80.9]	49.13 [41.5–56.8]	70.52 [63.1–77.2]
Specificity	45.08 [37.9–52.4]	78.24 [71.7–83.8]	42.49 [35.4–49.8]	43.52 [36.4–50.8]	60.62 [53.3–67.6]	54.92 [47.6–21.1]	56.99 [49.7–64.1]	72.54 [65.7–78.7]	73.58 [66.8–79.6]
LR + c	1.37	1.33	1.35	1.32	1.83	1.44	1.73	1.79	2.67
LR − d	0.55	0.91	0.53	0.58	0.46	0.64	0.45	0.70	0.40
OR	2.4131 [1.5–3.8]	1.49 [0.9–2.4]	4.42 [2.7–7.3]	2.32 [1.5–3.6]	4.11 [2.6–6.4]	2.25 [1.5–3.4]	1.58 [1.0–2.4]	1.75 [1.1–2.8]	2.68 [1.7–4.1]
*p* -Value OR	0.0001	0.0987	0.0001	0.0002	0.0001	0.0002	0.0313	0.0182	<0.0001
**Panel B** ***p*** **-Values for the difference between AUCs returned by pairs of algorithms**									
	TiC*Clinical ONLY	**F5L + F2**	F5L + F2+ TiC*Clinical ONLY	TiC*GRS ONLY	**TiC**	TiC*GRS ONLY + F11 + FGG	**TiC + F11 + FGG**	TiC*GRS ONLY MOD	**TiC MOD**
TiC*Clinical ONLY		0.1746	0.5302	0.6761	0.0001	0.2978	0.0061	0.0868	<0.0001
F5L + F2			0.0166	0.0056	<0.0001	0.0006	<0.0001	0.0006	<0.0001
F5L + F2 + TiC*Clinical ONY				0.8817	0.0291	0.3509	0.0108	0.1182	<0.0001
TiC*GRS ONLY					0.0320	0.0575	0.0104	0.0678	<0.0001
TiC						0.1086	0.4112	0.3765	0.0004
TiC*GRS ONLY + F11 + FGG							0.05	0.2986	<0.0001
TiC + F11 + FGG								0.2744	0.0006
TiC*GRS ONLY MOD									<0.0001

Abbreviations: AUC, area under the curve; BMI, body mass index; FGG, fibrinogen gamma gene; GRS, genetic risk score; PT, prothrombin; SNP, single-nucleotide polymorphism; TiC, Thrombo inCode; VTE, venous thromboembolism.

a AUC = area under the receiver operating characteristic curve (ROC curve).

b *p* -Value of the AUC.

c Positive likelihood ratio.

d Negative likelihood ratio.

Table 5 compares the results for the TiC score with the additional GRS and risk algorithms outlined in Table 2 . The use of the TiC clinical variables alone (TiC*Clinical ONLY) generated an AUC value comparable to that of F5L + F2 (0.576 vs. 0.537; *p* = 0.1746). The addition of these clinical variables to the GRS increased their AUCs significantly, except for the TiC*GRS-ONLY- + F11 + FGG combination.

The addition of F11 rs2036914 and FGG rs2066865 to the TiC score GRS did not improve its AUC (TiC*GRS-ONLY- + F11 + FGG vs. TiC*GRS-ONLY 0.608 vs. 0.588; *p* = 0.0575). Nor did it improve the AUC when these two variants were included in the TiC score algorithm (TiC + F11 + FGG vs. TiC 0.679 vs. 0.673; *p* = 0.4112). These observations were also similar to those obtained for the Spanish population. 12 As the control population was selected to have a similar family history than cases, TiC has demonstrated to be more useful than the family history to identify patients at high risk of VTE.

Table 6 shows that in both populations the allelic frequency of the risk alleles for blood type A1 was higher among the case subjects than the controls. The Swedish case subjects also showed a higher frequency for the risk alleles SERPINA10 rs2232698 and FGG rs2066865 compared with the controls. 12 In contrast, the Spanish cases had higher allelic frequencies for F12 rs1801020, F2 rs1799963, and the risk alleles in the gene for Factor V.

**Table 6:** Presence of risk alleles in cases and in controls in the Swedish and Spanish populations

	Swedish			Spanish		
Gen	Cases a	Controls a	*p* -Value	Cases a	Controls a	*p* -Value
	*n* = 173	*n* = 196		*n* = 248	*n* = 249	
ABO b	102 (58.96)	79 (40.31)	0.0004	147 (59.0)	87 (35.70)	<0.0001
F12	75 (43.35)	92 (46.94)	0.48899	15 (6.02)	5 (2.02)	0.0233
Serpin A10	4 (2.31)	0	0.0326	10 (4.02)	4 (1.61)	0.1045
Serpin C1	0	0		4 (1.61)	1 (0.40)	0.1765
F5 c	40 (23.12)	35 (17.86)	0.2108	32 (12.90)	5 (2.02)	<0.0001
F13	69 (39.88)	96 (48.98)	0.0798	146 (58.6)	139 (56.50)	0.6361
F2	12 (6.93)	7 (3.57)	0.1454	15 (6.02)	5 (2.02)	0.0233
F11	142 (82.08)	163 (83.16)	0.7848	162 (65.32)	120 (66.26)	0.8254
FGG	99 (57.22)	87 (44.39)	0.0140	98 (39.40)	92 (37.90)	0.7316

Abbreviation: FGG, fibrinogen gamma gene.

a With at least one risk allele. Expressed as *n* (%).

b With at least one allele for the A1 ABO subgroup.

c Only F5 Leiden was found.

Table 7 shows the general differences between the Swedish and Spanish populations. Among the case subjects and controls, the risk alleles in the gene for Factor V, F11 rs2036914, and F12 rs1801020 were more common among the entire Swedish population (i.e., cases plus controls) than the entire Spanish population. In addition, the Swedish case subjects showed a lower allelic frequency of F13 rs5985 and a higher frequency of the risk allele FGG rs2066865 than did the Spanish case subjects. 12

**Table 7:** Presence of risk alleles in case and control subjects belonging to the Swedish and Spanish populations

	Swedish	Spanish		Swedish	Spanish	
Gen	Cases a	Cases a	*p* -Value	Controls a	Controls a	*p* -Value
	*n* = 173	*n* = 248		*n* = 196	*n* = 249	
ABO b	102 (58.96)	147 (59.0)	0.9935	79 (40.31)	87 (35.70)	0.3198
F12	75 (43.35)	15 (6.02)	<0.0001	92 (46.94)	5 (2.02)	<0.0001
Serpin A10	4 (2.31)	10 (4.02)	0.3357	0	4 (1.61)	0.0747
Serpin C1	0	4 (1.61)	0.0940	0	1 (0.40)	0.3759
F5 c	40 (23.12)	32 (12.90)	0.0062	35 (17.86)	5 (2.02)	<0.0001
F13	69 (39.88)	146 (58.6)	0.0002	96 (48.98)	139 (56.50)	0.1149
F2	12 (6.93)	15 (6.02)	0.7076	7 (3.57)	5 (2.02)	0.3174
F11	142 (82.03)	162 (65.32)	0.0002	163 (83.16)	120 (66.26)	0.0001
FGG	98 (57.22)	98 (39.40)	0.0003	87 (44.39)	92 (37.90)	0.1671

Abbreviation: FGG, fibrinogen gamma gene.

a With at least one risk allele. Expressed as *n* (%).

b With at least one allele for the A1 ABO subgroup.

c Only F5 Leiden was found.

For the purpose of comparison, a modified TiC GRS (TiC*GRS-ONLY-MOD) and TiC algorithm (TiC*MOD) were studied, adjusting for the allelic frequencies of the Swedish population. The TiC*GRS-ONLY-MOD algorithm returned an AUC value with nonsignificant difference to that provided by the original TiC*GRS-ONLY model (0.636 vs. 0.588; *p* = 0.0678). The TiC*MOD algorithm returned an AUC value significantly higher than that provided by the original TiC (TiC*MOD vs. Ti 0.783 vs. 0.673; *p* = 0.0004) in the Swedish population.

Also, for the purpose of comparison and considering that of those genetic variants included in TiC, ABO variant was the most significantly present in VTE cases, we studied the performance of a new score. That formed by ABO, Factor V Leiden, and PT genetic variants. The score formed by ABO, Factor V Leiden, and PT genetic variants showed an AUC of 0.609, with a sensitivity of 69.39 and a specificity of 50.35. The AUC was significantly lower than that of TiC ( *p* = 0.0165).

## Discussion

The main problem in the prevention of VTE is the identification of those who are at serious risk. Evaluating risk factors for VTE is crucial when weighing up the risk of bleeding against that of a first VTE, recurring VTE, or obstetrical complications. It is well accepted that VTE is a multifactorial disease precipitated by a combination of clinical and genetic risk factors. However, accurately predicting a person's risk of developing a VTE is difficult.

Our group and others 12 17 18 have shown that clinical/GRS models for estimating the risk of VTE have better predictive capacity than the classical F5L + F2 combination. The TiC score has also shown clinical value in predicting VTE in patients with cancer, 19 and can be used to identify women with recurrent pregnancy loss in whom thrombophilia may be a contributing factor. 20

The use of a GRS might not always be generalizable across populations given differences in allelic frequencies. However, the present results show that the TiC score developed in population from Southern Europe reliably predicted VTE in a population from Northern Europe, despite significant differences in the allelic frequencies of the contemplated genetic variants. For both populations, the TiC score returned similar results, and always significantly better than the F5L + F2 combination. As reported earlier for the Spanish population, 12 no improvement was seen in the capacity of the TiC score (determined as either TIC-GRS*ONLY or TiC) when F11 rs2036914 and FGG rs2066865 were added to the algorithm.

The TiC*GRS-ONLY-MOD score yielded an AUC ROC value no better than that provided by the TiC*GRS-ONLY model ( Table 5 ). However, the TiC*MOD algorithm—which took into account the specific allelic frequencies of the Swedish population—increased the AUC to 0.783. This suggests that the TiC algorithm can be tailored to various populations to optimize its prediction of risk. Similar behaviors have been reported for other risk algorithms. For instance, the Framingham risk score, which predicts coronary events, can be used worldwide, but adaptations to local populations significantly improve its predictive ability. 21 22

In a similar manner to the concern that the magnitude and direction of allelic effects can differ between populations, the concern exists about the predictive capability of GRS scores in different ethnicities. We have studied in persons of African, Latino, and East-Asian ancestry the predictive capability of cardiovascular events of a GRS developed in Europeans. 23 We found that the GRS developed in Europeans provided similar results when used in other ethnicities. Wassel et al have studied GRS related to VTE in a multiethnic cohort. 24 They also conclude that the GRS had a similar capability among the different ethnicities. In none of those studies, an adapted GRS was studied.

The clinical utility of TiC is in the decision-making process, when the physician has to decide whether or not to initiate thromboprophylaxis in a patient. In this case, TiC has proved to be better than clinical variables and the classic F2 + F5 test in identifying the subjects a high risk of developing a VTE, and therefore in need of thromboprophylaxis. TiC can be taken as a predictive risk, because we identify subjects at high risk of developing VTE.

The present work suffers from the limitation that the number of subjects studied was relatively low. However, in its favor, the TiC score involves an algorithm that combines clinical and genetic variants that individually have been repeatedly associated with VTE in different populations.

In conclusion, the present results show the TiC score to predict the risk for VTE well, and to be better in this regard than the F5L + F2 combination. Further, they show that the TiC score can be used to reliably predict the risk of VTE despite differences in the allelic frequencies between populations. It can do this even better when modified to take into account the specific allelic frequencies of the population under study.

## Notes

**What Is Known about This Topic?**

- The two genetic variants (Factor V Leiden and 20210 G/A in PT gene) commonly used for VTE risk estimation have a very poor performance.
- A recently developed risk score TiC has been shown to have a higher risk estimation capability is southern European patients.

**What This Paper Add?**

- The risk estimation capability of TiC has been verified in Swedish patients.
- The higher risk estimation capability of TiC was preserved despite the difference in the frequency of the genetic variants between northern and southern European population.
- As happens with other risk estimation equations in cardiovascular field, the estimation capability of TiC can be improved if adapted to the specific country frequency of the genetic variants.
